# Abnormal Vision-Based Displacement Perception in Parkinson’s Disease

**DOI:** 10.3389/fnins.2021.676469

**Published:** 2021-07-26

**Authors:** Matthew Bernardinis, S. Farokh Atashzar, Rajni V. Patel, Mandar S. Jog

**Affiliations:** ^1^School of Biomedical Engineering, Western University, London, ON, Canada; ^2^Canadian Surgical Technologies and Advanced Robotics, London Health Sciences Centre (LHSC), London, ON, Canada; ^3^London Movement Disorders Centre, LHSC, London, ON, Canada; ^4^Electrical and Computer Engineering, Mechanical and Aerospace Engineering, New York University (NYU), New York, NY, United States; ^5^Department of Clinical Neurological Sciences and Electrical and Computer Engineering, Western University, London, ON, Canada

**Keywords:** Parkinson’s disease, perception, vision, displacement, levodopa, deep brain stimulation, *de novo*, non-motor

## Abstract

In this work, we investigate the effect of Parkinson’s disease (PD), and common corresponding therapies on vision-based perception of motion, a critical perceptual ability required for performing a wide range of activities of daily livings. While PD has been recognized as mainly a motor disorder, sensory manifestation of PD can also play a major role in the resulting disability. In this paper, for the first time, the effect of disease duration and common therapies on vision-based perception of displacement were investigated. The study is conducted in a movement-independent manner, to reject the shadowing effects and isolate the targeted perceptual disorder to the maximum possible extent. Data was collected using a computerized graphical tool on 37 PD patients [6 early-stage *de novo*, 25 mid-stage using levodopa therapy, six later-stage using deep brain stimulation (DBS)] and 15 control participants. Besides the absolute measurement of perception through a psychometric analysis on two tested position reference magnitudes, we also investigated the linearity in perception using Weber’s fraction. The results showed that individuals with PD displayed significant perceptual impairments compared to controls, though early-stage patients were not impaired. Mid-stage patients displayed impairments at the greater of the two tested reference magnitudes, while late-stage patients were impaired at both reference magnitudes. Levodopa and DBS use did not cause statistically significant differences in absolute displacement perception. The findings suggest abnormal visual processing in PD increasing with disease development, perhaps contributing to sensory-based impairments of PD such as bradykinesia, visuospatial deficits, and abnormal object recognition.

## Introduction

Although movement abnormalities have clinically defined Parkinson’s disease (PD) since its 19th century definition, the motor system is not necessarily the sole root of abnormalities (Jankovic, 2008). Accurate movements rely on the initial collection and processing of environmental information by the sensory nervous system, sensorimotor integration, and the production of motor output signals sent to muscles, with disruption of any system impairing movement (Singer, 1980; Noback et al., 2005). As exemplified with force computing and reproduction that is accurate in simple tasks, but slowed and variable during complex tasks, aspects of the abnormal motor functionality of PD can be rooted in neural dysfunction (Stelmach et al., 1989; Lafargue et al., 2008). Accordingly, movement abnormalities in PD are not necessarily due to abnormal motor function alone. Rather, motor dysfunction may be influenced by improper neural processing of stimuli, which along with PD-induced sensorimotor integrative deficiencies could lead to the observed motor dysfunction. However, due to movement generation being heavily used as a measure of the response in most clinical studies on PD it is not possible to decipher if abnormalities arise through the perceptual, motor, or sensorimotor integration deficits.

Visual information involving object and spatial properties is crucial for navigation and the production of accurate active and reactive movements (Azulay et al., 1999; Davidsdottir et al., 2005). Modern visual experience theory suggests vision to be a means to gain knowledge used to explore and manipulate the space around the host through motor activities (O’Regan and Noë, 2001). Considering this, vision perception and motion production are linked processes, with visual information processing being imperative for movement. Anatomically, visual perceptions are linked to a dorsal parietal stream involved in mapping an object’s location in space, and a ventral occipitotemporal stream involved in object identification and memory (Goodale and Milner, 1992; Horwitz et al., 1992).

Visuospatial abnormalities in memory and representation of three-dimensional space are common among individuals with PD (Davidsdottir et al., 2005). These impairments can contribute to balance and navigation deficits increasing fall risk and injury (Azulay et al., 1999; Wood et al., 2002; Davidsdottir et al., 2005). Although visually-related deficits are common characteristics of PD (Corin et al., 1972; Wright et al., 1990; Haug et al., 1994; Büttner et al., 1995; Adamovich et al., 2001; Barnes and David, 2001; Zhu et al., 2016; Joyce et al., 2018), there is no systematic investigation on processing abnormalities of visual information for tasks relevant to movement that are not confounded by motor impairment. Furthermore, past findings of object recognition and navigational impairments observed in PD (Wood et al., 2002; Laatu et al., 2004; Davidsdottir et al., 2005; Lawrence et al., 2007; Clark et al., 2008; Almeida and Lebold, 2010; Martens and Almeida, 2012; Nantel et al., 2012) indicate PD-induced impairments in ventral occipitotemporal visual processing may exist. Recently, we have designed a computerized movement independent task using a virtual reality environment to generate computational statistical models of visual perceptions of time, which showed deficits in the accurate discrimination of temporal durations for those with PD (Bernardinis et al., 2019). The current paper extends the use of the computerized module allowing for statistical understanding of vision-based displacement perception.

We propose that the perceptual “tuning” of individuals with PD may be distorted, leading to improper processing of perceptual stimuli, thus causing inappropriate motor output. Although this motor output may be what one might expect based on the perception (i.e., is congruent to the perception), it is still incorrect due to perceptual inaccuracy, exemplified in healthy individuals by the changing of one’s stride length and speed when perceiving a surface to be icy even if it eventually determined to be dry. To investigate this phenomenon, we must first assess the pure perceptual ability of PD patients. In this work, we have studied visual processing in PD through a perceptual task resembling movement-related displacement perception tasks (Demirci et al., 1997; Konczak et al., 2007), while isolating visual processing from movements and sensorimotor integration. Thus, we observed the ability of individuals with PD in accurately perceiving movement-independent visual displacement information. The impact of disease duration and the effect of dopaminergic and surgical treatment were also assessed, providing deeper insight on how the disease affects visual processes and the treatment effect on these abnormalities.

## Materials and Methods

### Experimental Design

**FIGURE 1:**
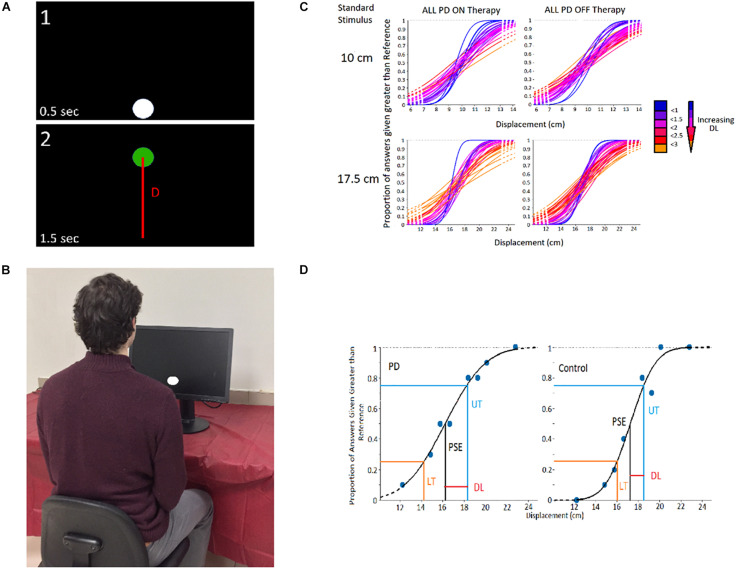
Experimental Design. **(A)** Visual Displacement Perception Trial — Participant compares displacement distance between circles (D; line not visible in the experiment). **(B)** Experimental Setup. **(C)** Cumulative Gaussian Distributions heat map of PD participants and controls, in which distributions more blue in colour signify a greater slope (thus greater participant perceptual sensitivity), and those more red/orange in colour signifying a lesser slope (and worse participant perceptual ability). **(D)** Example analysis of Cumulative Gaussian Distribution to obtain DL.

To study visual displacement perception independent of movement, a two-alternative forced-choice experiment (displayed in Figure 1A) was conducted. In the task, the displacement distance between two circular displacements presented in series is compared. Each displacement began with a white circle presented near the top or bottom of the monitor, followed by a displaced green circle. The participant responded (without time constraint) which displacement they perceived to be the largest in distance. In each of the 160 trials, one of two “standard stimuli” (10 and 17.5 cm) were compared to one of 8 “comparison stimuli.” The comparison stimuli magnitudes for the 10 cm standard stimulus were 7, 8.5, 9, 9.5, 10.5, 11, 11.5, and 13 cm; and the comparison magnitudes for the 17.5 cm standard stimulus were 12.25, 14.85,15.75, 16.62, 18.36, 19.25, 20.1, and 22.75 cm. Comparison values were chosen based on pilot testing of healthy adults, in which comparison stimuli differing in magnitude the most from the standards were always answered correctly, and those differing in magnitude the least were answered correctly 50% of the time.

### Testing Apparatus

Experimental visual stimuli were solely displayed on an LG Flatron W2242PM 22-inch visual monitor (resolution: 1,680 × 1,050). Participants sat in a comfortable, upright position approximately 60 cm (∼2 feet) in front of the monitor (Figure 1B). Both the height of the chair and monitor were adjusted for optimum viewing. Each participant, along with the examiner, were in an isolated room, minimizing auditory and visual distractions. The visual perception test was run in a virtual-reality environment designed at the Canadian Surgical Technologies and Advanced Robotics (CSTAR) lab and was connected to a real-time Matlab-Simulink program controlled by the experimenter.

### Participants

Thirty-seven patients with PD (30 males, seven females) and 15 healthy, age-matched controls (12 females, three males) with no known neurological or psychiatric disorders were recruited from the Movement Disorders Program at London Health Sciences Centre, University Hospital in London, Ontario, Canada (Table 1). Recruitment involved assessing potential participant enrollment eligibility based on cognitive aptitude, participants fitting into the early-stage *de novo*, mid-stage levodopa using, or later-stage DBS using subgroups (based on the progression of PD and therapy usage), and the exclusion of candidates exhibiting PD symptoms that would impair experimental assessments (such as inabilities to focus, excessive fatigue, dystonia, etc.). These eligible candidates were met during clinic visits, where the experiment was described and their ability to conduct the experiment was further assessed based on cognitive fitness. Control participants generally had relation to PD participants, often being a spouse, family member, or friend, and were also contacted during clinic visits. The study protocol for this work was approved by the Research Ethics Board of the University of Western Ontario (REB 107253). All participants provided informed consent for participation in the study. All experiments were performed in accordance with the Tri-Council Policy Statement of Ethical Conduct for Research Involving Humans in Canada, as well as the Declaration of Helsinki. Of the 37 PD patients, 25 were treated using levodopa medication daily (half-life: 1–1.5 h) (Brooks, 2008). Though individual equivalent dose of levodopa differed from patient to patient, most consumed 200 mg of levodopa 3–4 times a day. Prior to experimentation, PD patients refrained from taking levodopa for at least 12 h to achieve a clinically defined OFF state. These patients initially conducted the experiment OFF levodopa, after which they were administered 300 mg of levodopa (unless their regular dose was 100 mg or lower, in which case they were administered 200 mg) and performed the task again (ON phase). No participants displayed dyskinesia with this acute dose. The ON and OFF experiments were conducted on the same day, involving a mandatory break of an hour after levodopa administration. Motor symptoms were assessed ON and OFF levodopa using section 3 (motor sub-scale) of the Unified Parkinson’s Disease Rating Scale (UPDRS). To further augment the findings and report additional observations, a pair of small-sample patient groups (*n* = 6 for both groups) relating to later-stage deep brain stimulation (DBS) using patients and early-stage *de novo* patients not yet using any PD therapy at the time of experimentation were analyzed as “case studies.” The intention of these case studies was to observe potential trends regarding the visual displacement perception abilities of shorter and longer duration PD patients, and to observe the perceptual effects of DBS use. Due to the small sample sizes of these groups, the statistical tests act only as indicators of potential trends rather than a confirmation that differences are occurring between certain populations. If later-stage patients using DBS were also using levodopa, they refrained from using the medication 12 h prior to the experiment and throughout the day of testing. It should be noted that OFF DBS refers to OFF-OFF conditions (OFF stimulation and OFF dopaminergic medication), with ON DBS referring to ON-OFF conditions (ON stimulation, OFF dopaminergic medication). Prior to experimentation, DBS devices were turned OFF. After a 45-min waiting period, patients carried out the task in the same fashion as patients using levodopa. Experimentation occurred initially in the OFF-stimulation state, followed by the device being turned on and an hour break before experimentation in the ON state. Of note, the average age of later-stage patients using DBS was substantially lower than the levodopa patient group (Table 1). This is largely due to the presence of cognitive impairment in many elderly later-stage patients. Early-stage *de novo* PD patients (*n* = 6) only carried out the experiment once. Cognitive assessment of PD patients was conducted using the Montreal Cognitive Assessment (MoCA) (Nasreddine et al., 2005). Diagnostic assessments for visual acuity (using reading tasks and the Snellen eye chart), and smooth pursuit and saccadic eye movements were performed on all participants (PD and control) by an experienced clinician. PD patients were excluded from the study if they displayed visual, oculomotor, or substantial cognitive (MoCA < 25) impairments. Furthermore, PD patients experiencing visual hallucinations (PD-VH) or using PD medications other than levodopa were excluded from the study. It should be noted that patients included in the study did not exhibit severe impairments in color perception that would affect experimental performance.

**TABLE 1:** Summary of Demographic and Clinical Data for Tested PD Patients.

	Levodopa	DBS	*De novo*	Control
**Demographic data**				
Number (n)	25	6	6	15
Age (years)	70.04 ± 6.80	55.16 ± 8.89	74.17 ± 3.97	67.71 ± 8.82
Gender (m/f)	22/3	4/2	4/2	3/12
Total Years of Education	13.4 ± 4.36	13.33 ± 2.50	13.00 ± 1.67	13.76 ± 1.80
Years Since Diagnosis	6.88 ± 4.36	11.5 ± 4.04	3.12 ± 2.0	N/A
**Clinical data**				
MoCA (out of 30)	26.68 ± 2.17	26.67 ± 3.08	27.83 ± 2.14	27.23 ± 1.59
UPDRS motor sub-scale OFF Therapy	23.92 ± 6.69	34 ± 10.51	22.33 ± 7.91	N/A
UPDRS motor sub-scale ON Therapy	14.72 ± 6.07	22.33 ± 7.92	N/A	N/A
UPDRS motor subscale OFF vs. ON Difference	9.20 ± 5.09	21 ± 5.62	N/A	N/A

*UPDRS, Unified Parkinson’s Disease Rating Scale; MoCA, Montreal Cognitive Assessment.*

### Statistical Analysis

Initially, the correctness of patient responses was computed for each comparison value of a given standard stimulus. This data was then used to generate a probabilistic model (i.e., cumulative Gaussian distribution psychometric function) of the patient’s perceptual ability for assessment where increased slope and shift signify perceptual impairment (Figure 1C; Fründ et al., 2011). For this, the Psignifit 4.0 third party Matlab toolbox was used. The participants’ point of subjective equality (PSE) for both standard stimuli were calculated. The upper threshold (UT) and lower threshold (LT) were obtained through analysis of the psychometric function (Figure 1D), signifying the magnitude of displacement that was discerned from the standard stimulus 75% of the time (Gescheider, 2013). To assess an individual’s absolute perceptual sensitivity for vision-based displacement perception, a two-forced alternative-choice assessment comparing two linear displacements was carried out as described by G. Gescheider (2013). The Difference Threshold (DL; DL = PSE–LT or DL = UT–PSE) was the unit used to measure perceptual sensitivity, signifying the difference in magnitude necessary to differentiate a stimulus from the standard stimulus. A participant’s DL is inversely proportional to their perceptual sensitivity, with smaller DL’s indicating greater perceptual ability. In each trial of the experiment one of the two standard stimuli magnitudes was compared to a smaller or larger comparison stimuli based on the standard stimulus present. Datum points were considered outliers and omitted from analysis if they were 1.5 × Interquartile Range (IQR) above the third quartile, or 1.5 × IQR below the first quartile.

The paired two-tailed *t*-test was utilized to statistically assess perceptual differences based on patient therapeutic state, and independent samples two-tailed *t*-tests were used for comparisons between PD and control groups. Furthermore, perceptual linearity rooted in “Weber’s Law” (Baird and Noma, 1978) was analyzed to provide boosted sensitivity toward detecting potential abnormalities that may not have been observed through absolute assessment of the probabilistic models. Based on Weber’s law, the ratio between an individual’s DL and the amplitude of the standard stimulus is constant (Coren, 2003; Gescheider, 2013). The quantifiable value of Weber’s Law, Weber’s Fraction (WF), is defined as WF = DL/S, where S represents standard stimulus magnitude. Perceptions of healthy humans measured by WF have shown a strong linear relationship, following Weber’s Law.

## Results

### PD vs. Control Displacement Perception Findings

#### Part A

**FIGURE 2:**
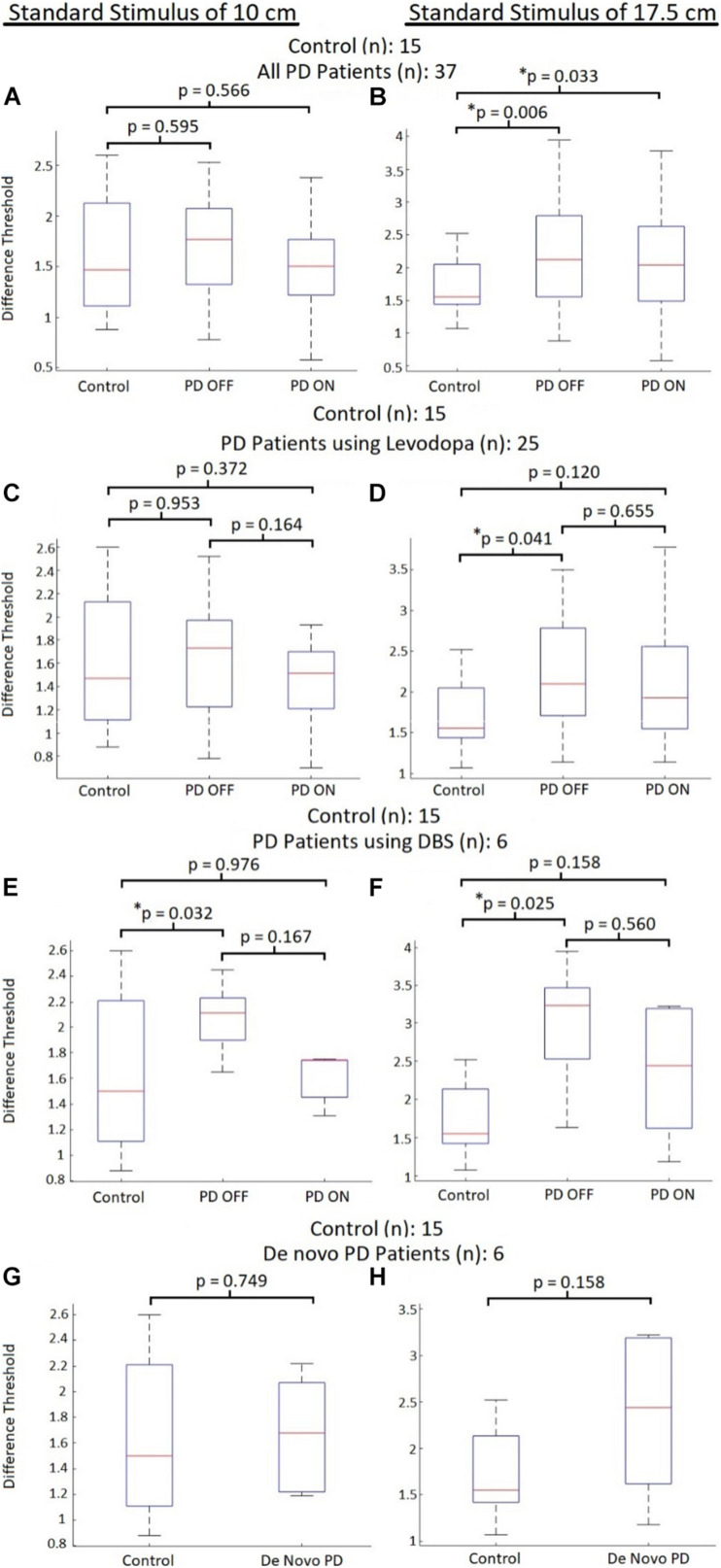
Control vs. PD absolute perceptual ability comparisons. Comparison of visual displacement perceptual abilities (quantified using DL) between control participants and PD patients subdivided into groups based on therapeutic treatment. Red lines represent median DL for each group, with bars representing the data spectrum. **(A)** Comparisons to all PD participants with the 10 cm standard. **(B)** Comparisons to all PD participants with the 17.5 cm standard. **(C)** Comparisons to PD participants using levodopa with the 10 cm standard. **(D)** Comparisons to PD participants using levodopa with the 17.5 cm standard. **(E)** Comparisons to PD participants using DBS with the 10 cm standard. **(F)** Comparisons to PD participants using DBS with the 17.5 cm standard. **(G)** Comparisons to de novo PD participants with the 10 cm standard. **(H)** Comparisons to de novo PD participants with the 17.5 cm standard.

When comparing all PD patients (those using levodopa, DBS, and *de novo* patients) with the control participants, there were no perceptual abnormalities seen at the smaller standard stimulus for those with Parkinson’s disease OFF their respective primary therapies (*p*-value = 0.595; average DL for PD patients OFF therapy: 1.69 ± 0.48; average DL for control participants: 1.60 ± 0.58) and ON their respective therapies [*p*-value = 0.566; average DL for PD patients ON therapy: 1.50 ± 0.47) (Figure 2A)]. However, for the larger tested standard stimulus of 17.5 cm, PD patients displayed significant impairments in visual displacement perception OFF their respective therapies (*p*-value = 0.006; average DL for PD patients OFF therapy: 2.22 ± 0.75; average DL for control participants: 1.70 ± 0.44), as well as significant impairments while ON their PD therapies (*p*-value = 0.033; average DL for PD patients ON therapy: 2.10 ± 0.78) (Figure 2B).

#### Part B

Focusing only on mid-stage PD patients using levodopa, for the standard stimulus of 10 cm, the average DL for PD patients OFF levodopa did not differ (*p*-value = 0.954) from the DL of control participants (average DL for patients OFF levodopa: 1.61 ± 0.49; average DL for control participants: 1.60 ± 0.58). This group of PD patients also displayed insignificant differences (*p*-value = 0.372) in their DLs when ON levodopa compared to the tested controls (average DL for patients ON levodopa: 1.41 ± 0.36) for the standard stimulus of 10 cm (Figure 2C). For the larger tested stimuli (compared to the 17.5 cm standard stimulus), the DLs of PD patients OFF levodopa were significantly greater (*p*-value = 0.041) than control participant DLs (average DL for patients OFF levodopa: 2.09 ± 0.68; average DL for control participants: 1.70 ± 0.44). In addition, there was no significant difference (*p-*value = 0.120) regarding greater DLs for PD patients ON levodopa compared to control participants (average DL for patients ON levodopa: 2.03 ± 0.79) (Figure 2D). Levodopa administration did not directly elicit any significant effects on the absolute perceptual sensitivity of displacement for PD patients. Regarding the standard stimulus of 10 cm, an insignificant trend (*p-*value = 0.164) toward reduced DLs was observed after the patients received levodopa (average DL of patients OFF levodopa: 1.61 ± 0.51; average DL of patients ON levodopa: 1.44 ± 0.56). In addition, for the standard stimulus of 17.5 cm there were no changes to average DL (*p-*value = 0.655) after the participants received levodopa (average DL of patients OFF levodopa: 1.95 ± 0.59; average DL of patients ON levodopa: 2.03 ± 0.79) (Figures 2C,D).

### Case Study A: Later-Stage Patients Using Deep Brain Stimulation

When looking at DLs between control participants and later-stage PD patients using DBS therapy at the standard stimulus of 10 cm, PD participants displayed on average significantly greater DLs (*p*-value = 0.032) than controls when OFF DBS (average DL for patients OFF DBS: 2.07 ± 0.29). However, when ON DBS, there was no difference in DLs (*p-*value = 0.976) between PD patients and controls (average DL for patients ON DBS: 1.61 ± 0.20) (Figure 2E). Regarding the standard stimulus of 17.5 cm, patients OFF DBS displayed significantly greater DLs (*p-*value = 0.025) compared to control participants (average DL for patients OFF DBS: 2.99 ± 0.86). At the larger standard stimulus, no differences in DLs (*p-*value = 0.158) were seen for PD patients ON DBS compared to controls [average DL for patients ON DBS: 2.35 ± 0.94) (Figure 2F)].

For the PD patients using DBS, no significant differences in DLs were seen between ON and OFF states. For the smaller tested stimulus magnitudes (compared to the 10 cm standard stimulus), there was no significant DL difference (*p-*value = 0.167) when patients were ON DBS (average DL of patients OFF DBS: 2.06 ± 0.17; average DL for patients ON DBS: 1.57 ± 0.21) (Figure 2E). At the larger tested magnitudes (standard stimulus 17.5 cm) there was again no difference in DL (*p-*value = 0.560) when the patients were using DBS (average DL for patients OFF DBS: 2.99 ± 0.86; average DL for patients ON DBS: 2.58 ± 0.37) (Figure 2F). It should be noted that a relatively strong trend toward reduced DLs was observed when participants were ON DBS (Figures 2E,F). However, due to the small sample size of the DBS PD group (*n* = 6), it is possible that the statistical analysis is not representative of the therapy’s impact on perceptual improvements. All but one of the patients using DBS displayed reduced DLs when ON DBS at both standard stimuli magnitudes.

### Effect of Levodopa vs. DBS

Although no direct comparison on an individual’s perceptual response to levodopa or DBS were made, comparisons of the therapies’ efficacy can still be inferred from the data. First, when comparing the UPDRS motor subsection scores of patients using levodopa to DBS users OFF their respective therapies (in their base PD state), we see DBS users have significantly greater (*p-*value = 0.006) UPDRS scores than levodopa users (average UPDRS section III score for DBS PD patients: 34.00 ± 10.50; average UPDRS section III score for levodopa PD patients: 23.92 ± 6.69) (Table 1). As expected, later-stage DBS users had significantly greater motor impairment compared to mid-stage PD patients using levodopa therapy. Similarly, when comparing the DL for the standard stimulus of 17.5 cm, DBS patients OFF therapy (mean DL: 2.99 ± 0.86) displayed significantly greater (*p-*value = 0.015) DLs than patients using levodopa (mean DL: 2.09 ± 0.68). Furthermore, at the standard stimulus of 10 cm, very substantial trends (*p-*value = 0.059) toward greater DLs in DBS patients (mean DL: 2.07 ± 0.29) were observed compared to levodopa only patients (mean DL: 1.61 ± 0.49). This again is to be expected based on the earlier mentioned findings, as later-stage PD patients displayed more severe impairment in the tested vision-based perception compared to mid-stage PD patients. However, when these patients were ON their respective therapies no significant differences were observed between DLs at both the 10 cm standard (*p-*value = 0.478; mean DL for DBS PD patients: 1.61 ± 0.20; mean DL for levodopa PD patients: 1.43 ± 0.52) and the 17.5 cm standard (*p-*value = 0.412; mean DL for DBS PD patients: 2.35 ± 0.94; mean DL for levodopa PD patients: 2.03 ± 0.79). These findings may suggest that DBS therapy has a greater efficacy in treating the vision-based perception of displacement when movement is not involved compared to levodopa.

### Case Study B: Early-Stage *de novo* Patients

Considering early stage *de novo* patients, for smaller stimuli magnitudes compared to the standard stimulus of 10 cm, there were insignificant differences between DLs (*p-*value = 0.749) of *de novo* PD patients compared to the control group (average DL for *de novo* patients was 1.68 ± 0.44) (Figure 2G). At the larger tested standard stimulus of 17.5 cm, *de novo* patients displayed an insignificant trend toward greater DLs (*p-*value = 0.158) compared to the control group (average DL for *de novo* patients: 2.35 ± 0.94) (Figure 2H). Thus, early-stage PD patients did not display significant differences in DL compared to controls.

### Displacement Perception Linearity

**FIGURE 3:**
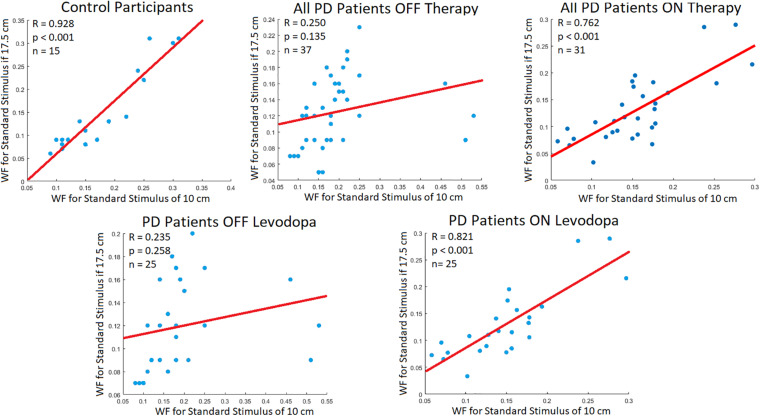
Participant WF Correlation. Correlations (R; Pearson correlation coefficient) between participant WF at the standard stimuli of 10 and 17.5 cm. The red line signifies the line of best fit for the correlation of data points. According to Weber’s Law, there should be strong correlations between WFs of different standard stimuli.

Indeed, this study agreed with Weber’s Law, showing a very strong correlation between the WF of the standard stimuli for healthy controls [Pearson correlation (*R*): 0.928, *p-*value < 0.001]. When comparing all PD patients OFF their respective therapies, they did not display significant correlations (*R* = 0.250, *p-*value = 0.135). However, when all PD patients were using their respective therapies, there were significant correlations seen between WFs (*R* = 0.762, *p-*value < 0.001). A similar pattern was observed when specifically looking at the levodopa group. For this group significant correlations were not observed when OFF levodopa (Pearson correlation: 0.235, *p-*value = 0.258). However, when these PD participants were administered levodopa strong correlations were observed between the WFs of different stimuli (Pearson Correlation: 0.821, *p-*value < 0.001) (Figure 3). Thus, administration of levodopa did appear to elicit some positive effects toward vision-based displacement perception.

## Discussion

This work shows that PD leads to visual, allocentric displacement perception impairments. These perceptual impairments arise without related movements, suggesting that the observed abnormality is intrinsic to the processing of visual information, and not dysfunctions occurring in sensorimotor integration or with the motor system. Although working memory and attentional deficit are well-noted symptoms of PD (Brown and Marsden, 1988; Calderon et al., 2001), it is improbable that these contributed to the observed perceptual abnormalities for the mid-stage PD patient group (using levodopa therapy) as no deficits were observed at the smaller tested magnitudes. Rather, the findings of the current study point toward impairment occurring in the ventral occipitotemporal and/or dorsal visual processing stream(s) in PD, showing a behavioral response to this known pathway. This provides a rationale regarding the potential basis for observed PD-induced deficits in activities utilizing ventral visual processing such as object and facial recognition (Laatu et al., 2004; Lawrence et al., 2007; Clark et al., 2008). This also is in similitude with the belief that freezing of gait (FoG) in PD is rooted in perceptual rather than motor deficiencies (Almeida and Lebold, 2010; Nantel et al., 2012). As proposed, the observed motor output may be an appropriate transformation of the instructions from the motor systems. However, the motor systems may be responding to errors in the processing and integration of visual information. Considering how this relates to FoG, errors in perception of distances processed through egocentric coordinates (using oneself as a reference) occurring in PD (Lee et al., 2001; Martens et al., 2013), as well as impairments in allocentric distances observed in the current study could lead to incorrect internal perceptions of one’s own dimensions and the dimensions of objects and structures in their surrounding environment. Thus, movement outputs are produced in relation to the skewed visual processing, for example, leading to errors in which an individual overestimates their size while underestimating the width of the doorframe, causing gait freezing.

When considering the effect of PD therapies, levodopa and DBS were shown to not directly increase perceptual sensitivity on the tested visual displacement task for either tested magnitudes. Though this agrees with prior work questioning dopamine’s relevance in movement-independent tasks impaired by PD and notions that common PD therapies are not beneficial for non-motor symptoms of the disease at their administered dosage (Deep-Brain Stimulation for Parkinson’s Disease Study Group, et al., 2001; Ahlskog, 2005; Chaudhuri et al., 2006), it is interesting nonetheless due to the current studies task (and visual processing in general) being tightly linked to movement processes. However, linear relationships between perceptual sensitivity and magnitude (as per Weber’s Law) were very weak when PD patients were OFF dopaminergic treatment, becoming strong when ON levodopa. We postulate this is due to dopaminergic treatment “tightening” the regulatory bounds of perception, in that the topology of the perceptual map re-orients after levodopa use, leading to greater overlap with controls in some domains. This would imply topographic perceptual maps are not normalized or “tightened” enough to allow all aspects of the perception to be improved. It is worth noting some aspects of non-motor disorders in PD do improve with levodopa use at the later-stages of the disease, specifically with the reduction of pain and anxiety (Fabbri et al., 2017).

Comparing patients using levodopa to those using DBS, we see that in OFF Parkinsonian states, the DBS-using participant group displayed significantly worse displacement perception abilities than levodopa-using patients. However, once these groups were administered their respective therapies, this gap in perceptual ability greatly shrank. This suggests that the improvements provided by subthalamic stimulation are greater than those brought on from dopaminergic treatment. However, it is possible that levodopa does indeed act in a beneficial manner toward correcting the visual displacement perceptual deficits in PD, and that it is other neurochemical imbalances (such as abnormal noradrenaline balances) or the widespread effect of levodopa targeting undesired neural regions that led to the observed perceptual abnormalities. It should be noted that a relatively strong trend regarding reductions in patient DL when ON DBS (compared to OFF DBS) was observed, along with impairments in subject DL (compared to controls) only being observed when OFF DBS (Figure 2). Thus, based on the results it appears that DBS might improve absolute visual displacement sensitivity for PD patients while levodopa did not cause improvement. However, statistical analysis was not fully representative of the population due to small power, necessitating future work for validation. If DBS does indeed display greater efficacy toward normalizing perceptual processing compared to dopaminergic therapies, it may support discussions and further investigations regarding the benefit of earlier surgical intervention.

The duration of PD appears to be related to performance in the tested allocentric visual perception task. Though the sample size is a limitation, *de novo* patients in the early stages of PD did not display any significant perceptual impairments in the current task compared to control participants. Patients in mid-stages of PD utilizing the dopaminergic medication as their primary PD therapy did display deficiencies in the displacement perception task. However, these were limited to the greater stimuli magnitudes compared to the standard stimulus of 17.5 cm, whereas late-stage patients (utilizing DBS therapy) displayed impairments at both tested standards. The results suggest that increased disease severity broadens the range of affected magnitudes. As tested individuals using DBS were at later stages of the disease, impaired memory and/or attentional performance might be involved (although subjects with observable deficits in these areas were rejected from study participation). Alternatively, increasingly severe PD symptoms may lead to a broader range of perceptual deficits through increasingly impaired occipitotemporal processing. This phenomenon should be further investigated as visual allocentric displacement perception may provide a valuable sensory modality that can be used to monitor PD progression without the use of motor function.

**FIGURE 4:**
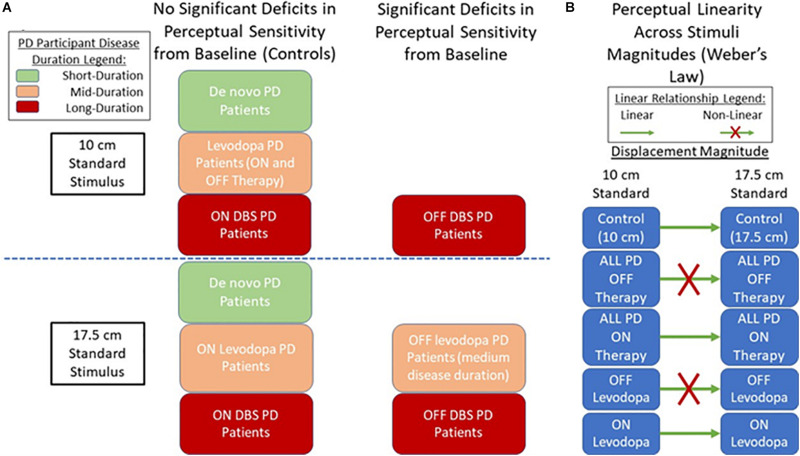
Displacement Perception in PD Summary. **(A)** Findings on displacement perception sensitivity between PD patient groups compared to control (baseline) participants. **(B)** Findings on the state of perceptual linearity according to Weber’s law.

To conclude our findings (Figure 4), allocentric visual displacement perception deficits independent of associated movements were observed in PD, with longer disease duration appearing to lead to more widespread perceptual abnormalities. Levodopa therapy did not appear to directly improve base perceptual ability; however, it may have modulated the parameters of perception to be more like controls (seen through improved perceptual linearity). DBS appeared to be more effective toward improving the studied perception, warranting further work analyzing its effect on non-motor perceptions. Future work should further investigate the neurological basis for these abnormalities and investigate the use of visual displacement perception for disease monitoring. Furthermore, future work should expand on the major limitations of the study, namely, the small sample sizes (particularly for the later-stage DBS using and early-stage *de novo* patient subgroups), and the discrepancy in the sex-makeup of the PD group (largely male) and control group (largely female) that may have impact on sex-based perceptual abilities (Herrera-Guzmán et al., 2004). The age discrepancy between later-stage patients using DBS and mid-stage patients using levodopa should be noted, along with the possibility of slight residual effects from levodopa occurring in participants using the treatment, though they are in a clinically defined OFF state.

## Data Availability Statement

The raw data supporting the conclusions of this article will be made available by the authors upon reasonable request.

## Ethics Statement

The studies involving human participants were reviewed and approved by Research Ethics Board of the University of Western Ontario (REB 107253). The patients/participants provided their written informed consent to participate in this study.

## Author Contributions

MB was involved in the conception and organization of the project, recruiting and execution of the research, statistical analysis of the data, and wrote the first draft and carried out further editing in subsequent drafts. SA contributed to the development of the research problem’s planning, execution, study design, and conduct, provided guidance for the experimental setup, and the statistical analysis for the project. RP proposed the research problem, contributed to the planning, organization, and development of the research project, and helped with the preparation and writing of the paper. RP and MJ supervised the thesis research of MB on which this manuscript is based. MJ assisted in the development of the clinical aspects of the research, and provided access to the participants included in the study, while also contributing to the analysis of the data and the preparing and writing of the manuscript. All authors have reviewed and edited the manuscript.

## Conflict of Interest

The authors declare that the research was conducted in the absence of any commercial or financial relationships that could be construed as a potential conflict of interest.

## Publisher’s Note

All claims expressed in this article are solely those of the authors and do not necessarily represent those of their affiliated organizations, or those of the publisher, the editors and the reviewers. Any product that may be evaluated in this article, or claim that may be made by its manufacturer, is not guaranteed or endorsed by the publisher.
